# Neural stem cell therapy in conjunction with curcumin loaded in niosomal nanoparticles enhanced recovery from traumatic brain injury

**DOI:** 10.1038/s41598-022-07367-1

**Published:** 2022-03-04

**Authors:** Abdolreza Narouiepour, Alireza Ebrahimzadeh-bideskan, Ghadir Rajabzadeh, Ali Gorji, Sajad Sahab Negah

**Affiliations:** 1 Department of Anatomy and Cell Biology, Faculty of Medicine, Mashhad University of Medical Sciences, Pardis Campus, Azadi Square, Mashhad, Iran; 2 Biomedical Research Center, Mashhad University of Medical Sciences, Mashhad, Iran; 3 Department of Food Nanotechnology, Research Institute of Food Science and Technology, Mashhad, Iran; 4 Shefa Neuroscience Research Center, Khatam Alanbia Hospital, Tehran, Iran; 5 Department of Neurosurgery, Westfälische Wilhelms-Universität, 48149 Munster, Germany; 6 Department of Neurology with Institute of Translational Neurology, Westfälische Wilhelms-Universität, 48149 Munster, Germany; 7 Epilepsy Research Center, Westfälische Wilhelms-Universität Münster, 48149 Munster, Germany; 8 Neuroscience Research Center, Mashhad University of Medical Sciences, Mashhad, Iran; 9 Department of Neuroscience, Faculty of Medicine, Mashhad University of Medical Sciences, Mashhad, Iran

**Keywords:** Cell biology, Neuroscience

## Abstract

Despite a great amount of effort, there is still a need for reliable treatments of traumatic brain injury (TBI). Recently, stem cell therapy has emerged as a new avenue to address neuronal regeneration after TBI. However, the environment of TBI lesions exerts negative effects on the stem cells efficacy. Therefore, to maximize the beneficial effects of stem cells in the course of TBI, we evaluated the effect of human neural stem/progenitor cells (hNS/PCs) and curcumin-loaded niosome nanoparticles (CM-NPs) on behavioral changes, brain edema, gliosis, and inflammatory responses in a rat model of TBI. After TBI, hNS/PCs were transplanted within the injury site and CM-NPs were orally administered for 10 days. Finally, the effect of combination therapy was compared to several control groups. Our results indicated a significant improvement of general locomotor activity in the hNS/PCs + CM-NPs treatment group compared to the control groups. We also observed a significant improvement in brain edema in the hNS/PCs + CM-NPs treatment group compared to the other groups. Furthermore, a significant decrease in astrogliosis was seen in the combined treatment group. Moreover, TLR4-, NF-κB-, and TNF-α- positive cells were significantly decreased in hNS/PCs + CM-NPs group compared to the control groups. Taken together, this study indicated that combination therapy of stem cells with CM-NPs can be an effective therapy for TBI.

## Introduction

Traumatic brain injury (TBI) is defined by a mechanical force to the brain tissue that induces cellular injury and triggers a variety of molecular dysfunctions^1^. TBI is a crippling disease that causes mortality and morbidity in developing countries, especially Iran^2,3^. Depending on the injury severities (i.e., mild to severe), patients usually suffer from long-term disabilities^4^. The pathological mechanisms of TBI can be classified into primary and secondary injuries^5^. Primary injury is caused by a mechanical force that leads to a variety of changes in the injury site, such as axonal destruction, contusion, laceration, and hematoma. Secondary injury takes place over a period of hours to days after primary insult. Secondary injury may result from impairment of the blood–brain barrier (BBB) and homeostasis that they increase the complications in cellular and molecular changes in the brain^6^. Among different mechanisms involved in TBI, neuroinflammation links primary injury to secondary injury and is likely a driver of chronic progressive neurodegeneration^7^.

The initial inflammatory signaling pathway after TBI stimulates microglia and astrocytes to migrate to the lesion area^8^. Microglia and astrocytes express different types of pattern recognition receptors (PRRs) that allow them to undergo an immune response^9^. Toll-like receptors (TLRs) are a class of intramembranous PRRs that play important roles in innate immune responses^10^. Among them, TLR4 has been widely recognized as the recognition of danger-associated molecular patterns (DAMPs) released by injured and necrotic cells within lesion areas^11^. The interaction between TLR4 and DAMPs stimulates the activation of a robust proinflammatory response in the course of TBI^5^. TLR4 has two adaptor proteins including myeloid differentiation primary response gene 88 (MyD88-) dependent pathway and MyD88-independent pathway (TRIF-dependent pathway)^12^. Stimulation of TLR4 recruits MyD88 and triggers NF-*κ*B that subsequently induces proinflammatory cytokines, such as interleukin-1β (IL-1β), interleukin-6 (IL-6), and tumor necrosis factor α (TNF-α)^13^. The TLR4/NF-*κ*B signaling pathway plays a crucial role in the pathogenesis of neuroinflammation after TBI^14^. Therefore, modulating the TLR4/NF-*κ*B signaling pathway can be regarded as a therapeutic option for treating TBI.

During the last decades, stem cell therapy has emerged as an innovative approach to treating the neuroinflammation of TBI^15^. Recent studies have shown that stem cells improve the functional recovery after TBI through immunomodulatory and regenerative properties^16^. Among different types of stem cells, numerous studies have demonstrated that neural stem cells (NSCs) transplantation has great neuroprotective properties that support functional recovery after acute TBI by mitigation of neuroinflammation and by the promotion of regenerative processes (i.e., increase neurogenesis, angiogenesis, and plasticity)^17–19^. NSCs are multipotent cells that can differentiate into neural lineages cells, such as neurons, astrocytes, and oligodendrocytes^20,21^. However, the low-transplanted NSCs survival rate remains challenging^22^. The inflammatory milieu is one of the main factors that have negative effects on the NSCs survival rate within the injury site. Therefore, manipulation of the inflammatory milieu to minimize the negative effects and maximize the beneficial effects of stem cells is necessary^23^. As a result, in terms of inflammatory milieu impacts on NSCs survival rate, stand -alone NSCs transplants may not be sufficient to repair properly injured tissue, while applying adjuvant therapeutics with immunomodulatory properties may be required^24^. Moreover, improving delivery into the brain and retention therapeutics in the brain should be mentioned^25^. Thus, encapsulation of therapeutics within nanoparticles (NPs) is one of the approaches that can improve site-specific delivery and bioavailability^26^. In this regard, the aim of this study was to investigate human NSCs transplant in a TBI model with nanoparticles containing anti-inflammatory agents. To this point, due to the anti-inflammatory and neuroprotective properties of curcumin, the major active component of turmeric, this component was selected as an adjuvant agent in this study^27,28^. Moreover, to improve the stability and permeability of curcumin into brain tissue, we used curcumin-loaded niosome nanoparticles (CM-NPs)^29^. Here, we show for the first time that combining NSCs derived from the human fetal brain with CM-NPs has the potential to improve functional recovery and reduce neuroinflammation in a TBI model by mitigating TLR4/NF-*κ*B pathway.

## Results

### Characterization of stem cells

**Figure 1:**
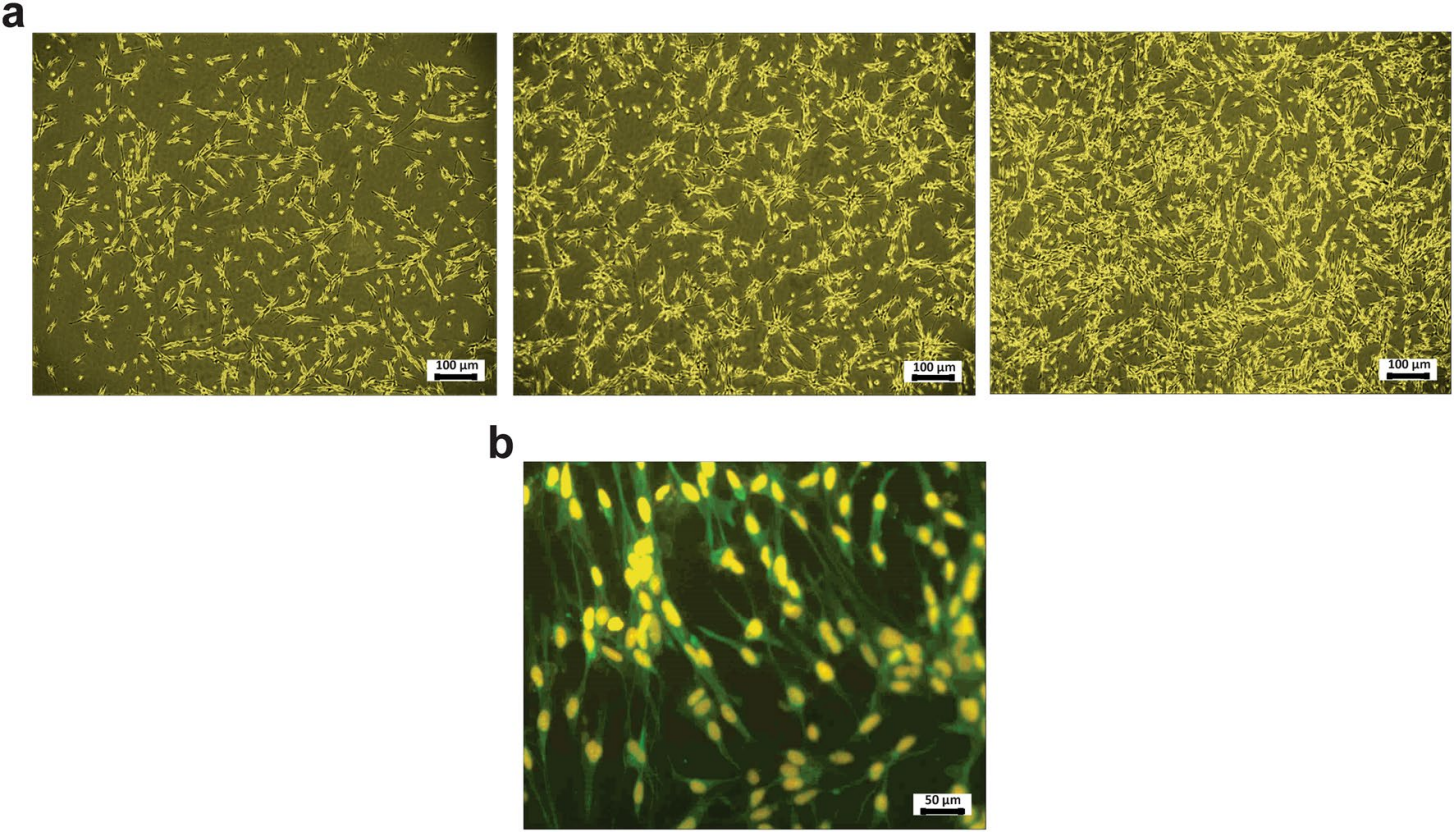
Culture and characterization of neural stem/progenitor cells (NS/PCs) derived from a human fetus. (**a**) Phase-contrast images of hNS/PCs on 5–7 days. hNS/PCs at 70–80% confluency were also shown. (**b**) Immunocytochemistry of hNS/PCs. Nestin as a neural stem cell marker was conjugated with FITC and nuclei were stained with PI.

The NS/PCs cells derived from the human fetal brain had a great potential to proliferate rapidly (Fig. 1a). In our study, an immunofluorescence assessment was performed to assess the expression of nestin as a neural stem cell marker in the hNS/PCs. Our results showed that the majority of hNS/PCs expressed nestin (Fig. 1b).

### Characterization of CM-NPs and detection of CM-NPs in brain

**Figure 2:**
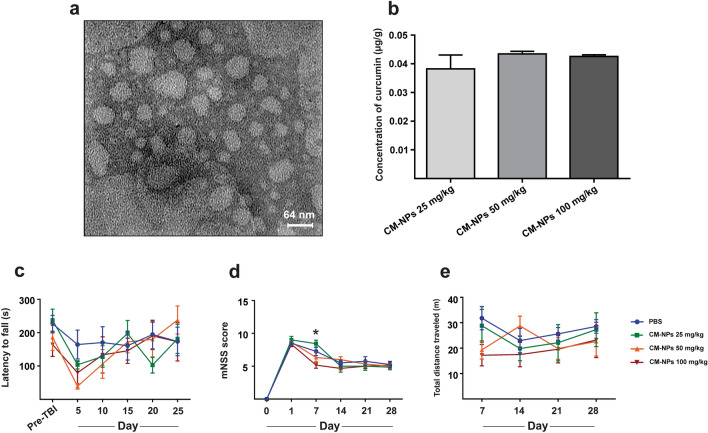
Characterization of CM-NPs. (**a**) Transmission electron microscopy (TEM) image of CM-NPs showed particles with spherical morphology and an average size of 60 nm. (**b**) The HPLC system was used to evaluate the ability of penetration of CM-NPs into the brain tissue. The representative micrograph shows different concentrations of curcumin in nanoparticles (i.e., 25, 50, and 100 mg/kg; n = 3). To find out an optimum dose of CM-NPs, some behavioral assessments, such as rotarod, mNSS, and OF were performed to evaluate the advanced locomotor function, sensory-motor function, and general loco-motor activity, respectively. (**c**) The rotarod test was performed before TBI as well as during predetermined time points after TBI. There was no significant difference for mean latency to fall between groups in the rotarod test. (**d**) The mNSS test was performed before TBI and predetermined time points after TBI in different groups. The mNSS score in the treated group with 50 mg/kg of CM-NPs was significantly decreased compared to 25 mg/kg of CM-NPs on day 7 after TBI. (**e**) Evaluation of total distance traveled in the open field in different groups on days 7–28 after TBI. The statistical analysis showed no significant difference for total distance traveled in the OF between groups. Data represented as the mean ± SEM and * indicates *P* < 0.05 (n = 6).

As shown in Fig. 2a, the structure of the CM-NPs was visualized by TEM. As can be seen, the nanoparticles are spherical and have a diameter in the range of 60–90 nm (Fig. 2a). In this study, HPLC was used to detect the presence of CM-NPs in brain tissue. It can be seen from the data in Fig. 2b that three different doses of CM-NPs were detected in brain tissue. The concentrations of CM-NPs in the brain for 25 mg/kg, 50 mg/kg, and 100 mg/kg were 0.382 ± 0.004, 0.434 ± 0.004, and 0.425 ± 0.004 μg/g, respectively (Fig. 2b).

### The optimum concentration of CM-NPs

To find out the optimal concentration of CM-NPs, several behavioral assessments, such as rotarod, mNSS, and OF tests were performed in a 28-day period according to the main study design in all TBI-induced rats. One day after the injury, animals randomly received orally either CM-NPs (25, 50, and 100 mg/kg/day, n = 9) or vehicle (n = 9). On the 25th day after the brain injury, the results indicated that the treated group with 50 mg/kg of CM-NPs stayed longer (237.83 s) on the rod than the treated group with 25 mg/kg of CM-NPs (181.57 s) and vehicle group (173.62 s) but in the rotarod test, there was no significant difference for mean latency to fall on the rod between groups (Fig. 2c). In addition, the mNSS score in the treated group with 50 mg/kg of CM-NPs was significantly decreased compared to 25 mg/kg of CM-NPs on day 7 after TBI (Fig. 2d; *P* < 0.05). Also, the statistical analysis showed no significant difference for total distance traveled in the open field between groups (Fig. 2e).

### Brain water content

**Figure 3:**
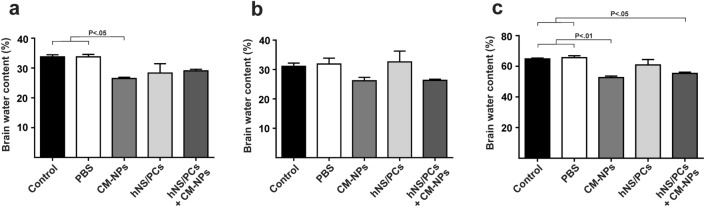
Brain water content was measured at 72 h after TBI in different groups. (**a**) Data from ipsilateral hemispheres have been shown that the CM-NPs group had a significantly lower brain water content than that of the control and PBS groups. (**b**) The statistical analysis showed no significant difference in brain water content in the contralateral hemispheres between groups. (**c**) The brain water content in the total cerebrum was significantly reduced in CM-NPs and hNS/PCs + CM-NPs treatment groups compared to the control and PBS groups. Data are presented as the mean ± SEM (n = 3).

To investigate the effect of therapeutic interventions on brain edema, we measured the water content of the brain in the affected or ipsilateral hemisphere, intact or contralateral hemisphere, and total cerebrum separately in all experimental groups. Increasing water content directly causes brain edema, which is one of the most important reasons for worsening brain damage and reaches its peak within 72 h after brain injury^30^. Our results showed that CM-NPs had the potential to reduce brain edema. As shown in Fig. 3a and c, the brain water content in the ipsilateral hemisphere and total cerebrum were significantly reduced in the CM-NPs group compared to the control and PBS groups (*P* < 0.05 and *P* < 0.01, respectively). We also observed a significant reduction of brain water content in the total cerebrum in the hNS/PCs + CM-NPs group compared to the control and PBS groups (*P* < 0.05), it can be said that treatment with CM-NPs plays an effective role in reducing brain edema (Fig. 3).

### Neurological assessments

**Figure 4:**
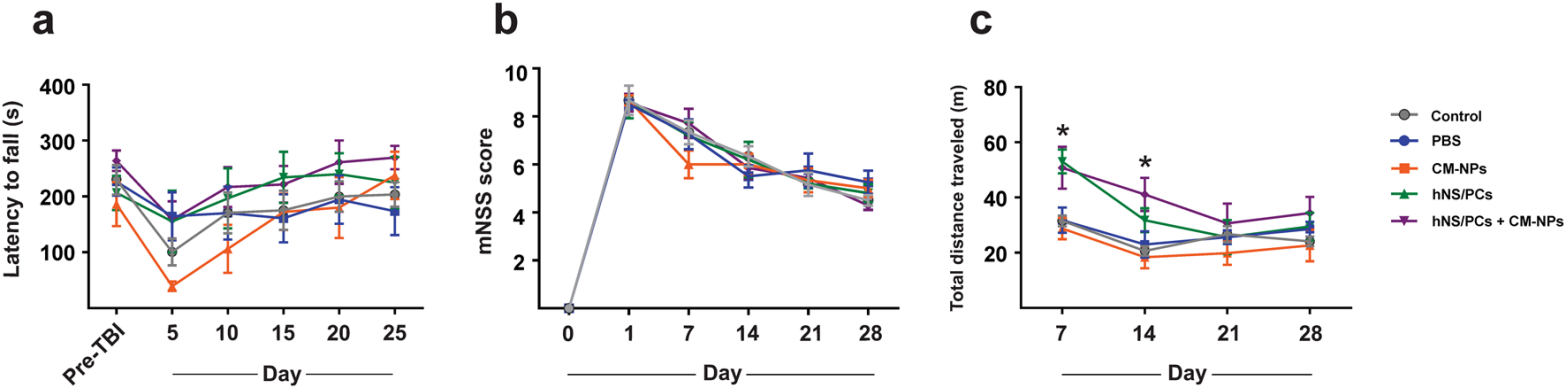
Rotarod, mNSS, and OF were assessed to evaluate the advanced locomotor function, sensory-motor function, and general locomotor activity in different groups following TBI. (**a**) The mean latency to fall on the rod showed no significant difference between groups in the rotarod test. (**b**) The statistical analysis showed no significant difference in mNSS scores between groups. (**c**) In the OF task, total distance traveled significantly increased in hNS/PCs and hNS/PCs + CM-NPs groups in comparison with the control, PBS, and CM-NPs groups on days 7 and 14. The data are shown as the mean ± SEM and * indicates *P* < 0.05 (n = 6).

To determine the long‑term weight support and hind-limb coordination, the rotarod test was used. The mean latency to fall on the rod showed no significant difference between groups in the rotarod test (Fig. 4a).

To assess the sensory-motor functions, the mNSS test was performed. The statistical analysis showed no significant difference in mNSS scores between groups (Fig. 4b).

To determine the general locomotor activity level, the OF test was performed. In the OF test, we observed that total distance traveled was significantly increased in stem cell groups (i.e., hNS/PCs and hNS/PCs + CM-NPs groups) compared to the other groups (Fig. 4c; *P* < 0.05).

### Brain lesion volume

**Figure 5:**
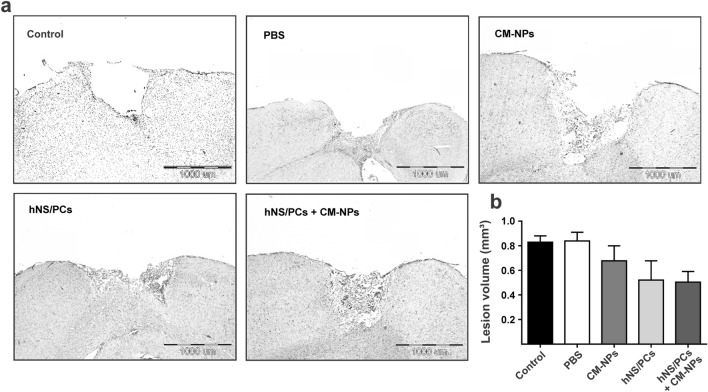
The mean lesion volume at the injury site after TBI in different groups. (**a**) Lesion areas are stained by hematoxylin–eosin (HE) in different groups. (**b**) The bar graph indicates the lesion volume in different experimental groups on day 28 after TBI. Data represented as lesion volume mean ± SEM (n = 6).

As indicated in Fig. 5a, the cavity of the injured area was approximately filled when transplanted stem cells. However, the statistical analysis showed no significant difference for this decrease in brain lesion volume (Fig. 5b).

### Neuroinflammation

#### Astrogliosis and reactive microglia

**Figure 6:**
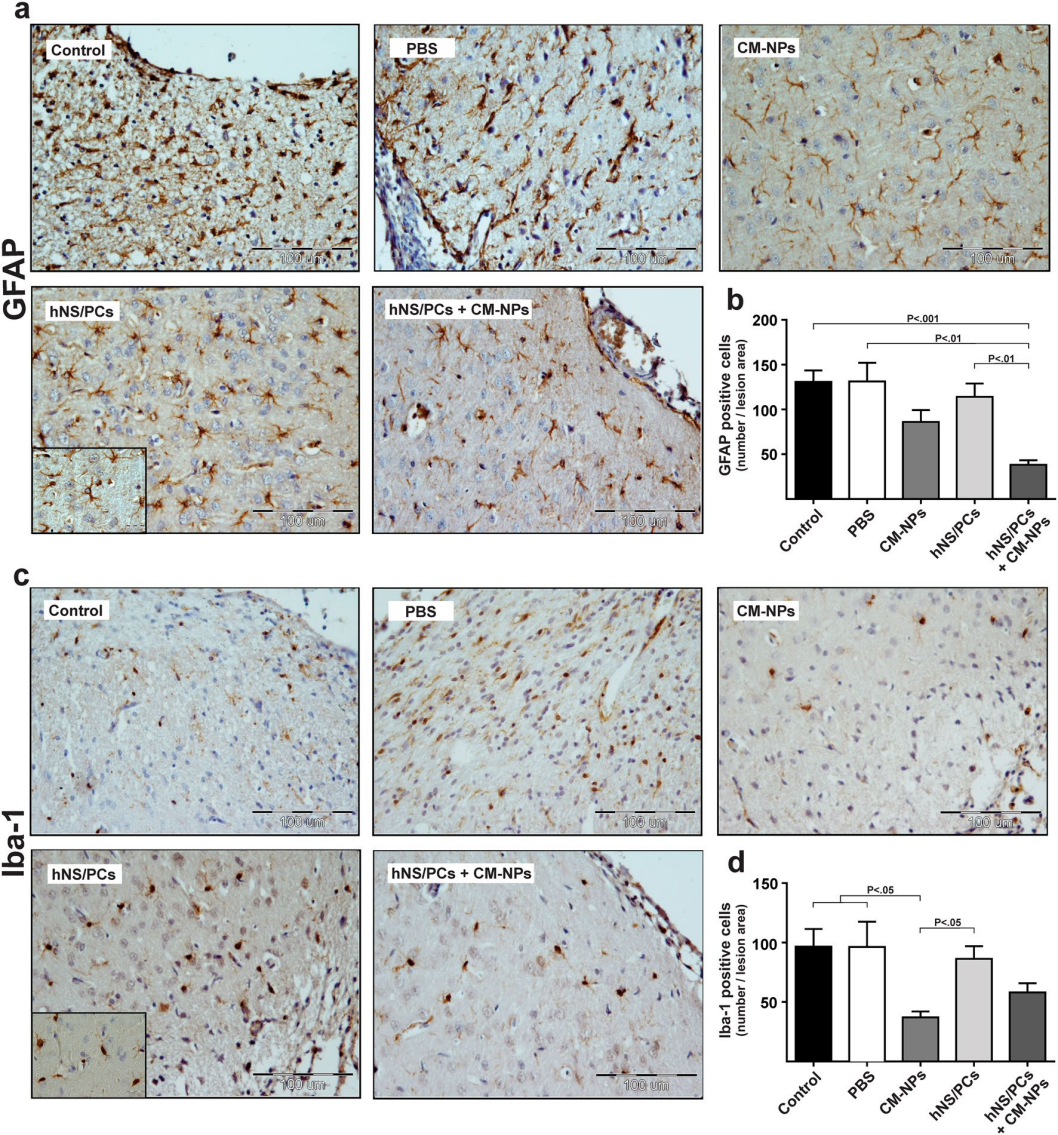
Immunohistochemistry staining of GFAP- and Iba-1-positive cells at the injury site after TBI and cell transplantation in rats. (**a**) GFAP-positive cells indicated brown stains are shown within the injury site after 28 days of TBI. (**b**) The mean number of GFAP-positive cells significantly decreased in the hNS/PCS + CM-NPs group compared to the control and PBS groups. Furthermore, the mean number of GFAP-positive cells in the hNS/PCS + CM-NPs group was significantly lower than the hNS/PCs group. (**c**) Immunohistochemistry slides of Iba-1-positive cells as indicated brown stain. (**d**) Bar graphs show the mean number of Iba-1-positive cells in the lesion area after 28 days of TBI in different study groups. The bar graphs indicate a significantly lower expression of Iba-1-positive cells in the CM-NPs group than in the control and PBS groups. Moreover, the mean numbers of Iba-1-positive cells in the CM-NPs group were significantly lower than that of the hNS/PCs group. Data are expressed as the mean ± SEM (n = 6).

To illustrate the therapeutic effect of hNS/PCs with CM-NPs on astrogliosis and reactive microglia in the injured brain, the expression of GFAP and Iba-1 in the lesion area were evaluated. After the brain injury, GFAP-positive astrocytes (Fig. 6a) and Iba-1-positive microglia (Fig. 6c) were observed in the lesion site. The mean number of GFAP-positive cells around the injury site was significantly decreased in the treated group with hNS/PCs + CM-NPs compared to the control (*P* < 0.001), PBS (*P* < 0.01), and hNS/PCs (*P* < 0.01) groups (Fig. 6b). Furthermore, the mean number of Iba-1-positive cells was significantly decreased in the CM-NPs group compared to the control (*P* < 0.01), PBS (*P* < 0.05), and hNS/PCs (*P* < 0.05) groups (Fig. 6d).

### TLR4/NF-κB/TNF pathway

**Figure 7:**
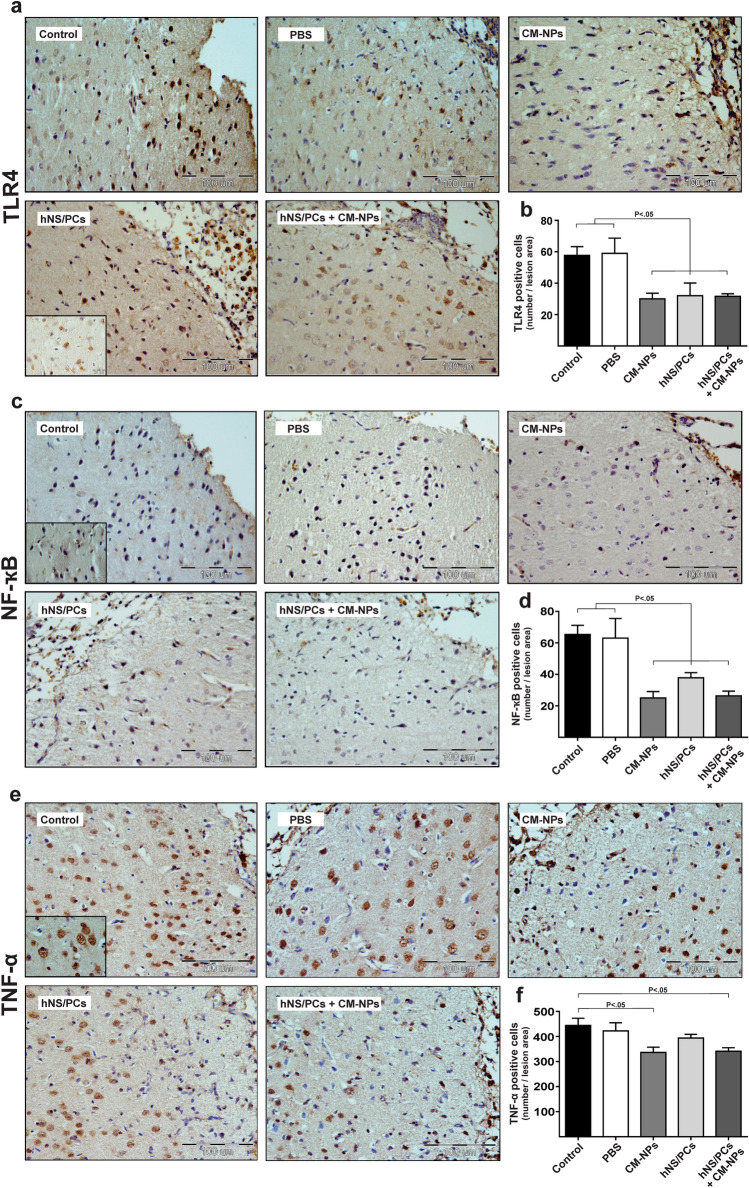
Immunohistochemistry staining of TLR4-, NF-κB-, and TNF-α-positive cells at the injury site after TBI and cell transplantation in experimental groups. (**a**) Representative images of TLR4-positive cells (i.e., brown stain) within the injury site on day 28 after induction of TBI. (**b**) The mean number of TLR4-positive cells significantly decreased in the CM-NPs, hNS/PCs, and hNS/PCS + CM-NPs groups compared to the control and PBS groups. (**c**) Representative images of immunohistochemistry slides of NF-κB-positive cells as shown brown stain. (**d**) Bar graphs show the mean number of NF-κB-positive cells in the lesion area after 28 days of TBI in different experimental groups. The bar graphs indicate a significantly lower expression of NF-κB-positive cells in the CM-NPs, hNS/PCs, and hNS/PCS + CM-NPs groups compared to the control and PBS groups. (**e**) TNF-α-positive cells indicated brown stains are shown within the injury site after 28 days of TBI. (**f**) The mean number of TNF-α-positive cells significantly decreased in the CM-NPs and hNS/PCS + CM-NPs groups compared to the control group. Data are presented as the mean ± SEM (n = 6).

To demonstrate the potential anti-inflammatory effects of hNS/PCs with CM-NPs, the expression of TLR4 (Fig. 7a), NF-κB p65 (Fig. 7c), and TNF-α (Fig. 7e) in the lesion area was evaluated. The TLR4-positive cells were significantly decreased in CM-NPs, hNS/PCs, and hNS/PCs + CM-NPs groups compared to the control and PBS groups (Fig. 7b; *P* < 0.05). In addition, the mean number of NF-κB-positive cells was significantly decreased in CM-NPs, hNS/PCs, and hNS/PCs + CM-NPs groups compared to the control and PBS groups (Fig. 7d; *P* < 0.05). Furthermore, to prove the role of the TLR4/NF-κB signaling pathway in response to our interventions, TNF-α expression in the lesion area was examined. The mean number of TNF-α-positive cells was significantly decreased in CM-NPs and hNS/PCs + CM-NPs groups compared to the control group (Fig. 7f; *P* < 0.05).

## Discussion

Our findings showed that significant neuroprotective effects were seen in the hNS/PCs + CM-NPs treatment group compared to the control groups in terms of “general locomotor activity” after brain injury. Brain edema also showed significant improvement in the hNS/PCs + CM-NPs treatment group compared to all other groups. Furthermore, a significant decrease in astrogliosis was seen in hNS/PCs + CM-NPs treatment group compared to other groups. However, no significant differences in activated microglia-, TLR4-, and NF-κB-positive cells were seen between the hNS/PCs + CM-NPs and CM-NPs groups. This result might be due to a beneficial effect of the CM-NPs itself, because studies have shown that CM-NPs can ameliorate neuroinflammation after injury by inhibiting stress oxidative and down-regulating NF-κB^31–33^. Overall, our study offers nanoparticle-based delivery as a potential opportunity to improve the environment of stem cells at the lesioned site.

During the last decades, the defects and disabilities following TBI remain one of the most important public health issues and concerns of communities around the world^34^. Brain edema formation is a principal aspect of TBI and is contemplated as a major independent risk factor for poor outcomes after TBI that indicates BBB disruption. BBB disruption in the course of TBI is an early event and peaks within hours after injury. From a cellular view of brain edema, astrocyte swelling (cytotoxic edema) characterizes the main constituent of the brain edema in the initial phase of TBI^35^. Evidence of BBB permeability and astrocyte swelling after TBI in animal models is characterized by the infiltration of serum into brain tissue that leads to neuroinflammatory events and apoptosis^35,36^. Our results demonstrated that the combination of hNS/PCs and CM-NPs significantly decreased TBI-induced brain edema and the number of reactive astrocytes. Our findings strongly suggest that transplantation of neural stem cells with CM-NPs can be used as an excellent approach to overcome the inhibitory effects of reactive astrocytes as a key element in the brain edema that occurs after TBI. These results reflect those of Ormond et al.^37^ who also found that curcumin in conjunction with stem cell therapy synergistically develops recovery from severe spinal cord injury.

As mentioned in the literature review, in a moderate to severe TBI, primary injury often leads to secondary consequences following inflammatory events. Microglia are the major players in initiating the inflammatory response after TBI^38,39^. Microglia are activated by the innate immune compartments (e.g., TLRs) and contributed to neurobehavioral function, neuroinflammation, brain edema, and gliosis^40,41^. In accordance with the beneficial and immunomodulatory effects of curcumin on microglia in previous studies^42,43^, a significantly lower number of reactive microglial cells (i.e., Iba-1-positive cells) was detected in the CM-NPs group than that of stem cell recipients groups. However, there were no significant differences between the combined treatment group with stem cells and CM-NPs groups. Our results are in contrast to the expectations and earlier findings^44,45^.

Activated astrocytes and microglia induce detrimental neurotoxic effects by releasing pro-inflammatory cytokines, such as IL-1β, IL-6, and TNF-α^46^. The most important regulators mediating the inflammatory cascade are the Toll-like receptors (TLRs) that can be activated by DAMPs followed by cellular injury products^47^. TLR4 as a pathogen recognition receptor (PRRs) is expressed by both microglia and astrocytes that activate intracellular signaling cascades in the inflammatory responses^48^. Nuclear factor κB (NF-κB) is targeted by TLR4 and translocates from the cytoplasm to the nucleus to regulate the expression of a series of inflammatory cytokines^13,49^. Previous studies have been revealed that TLR4/NF-κB signaling pathway is activated during the progress of post-TBI secondary injury; therefore, the suppression of the TLR4/NF-κB signaling pathway is a target option for TBI^50–52^. The application of curcumin and exosomes derived from stem cells after CNS injuries have been detected as an anti-inflammatory potential by inhibiting TLR4/NF-κB and down-regulating of inflammatory cytokines^14,27,53,54^. These results are in accord with our findings indicating that the expression of TLR4-, NF-κB, and TNF-α-positive cells were significantly decreased in monotherapy-treated rats. Overall, our results suggest that nanoparticles containing curcumin can ameliorate the microenvironment for stem cell effects. Our findings are in agreement with recent studies indicating that the nano-form of curcumin can improve the neuroprotective efficacy due to the high stability and high permeability of curcumin^32,55^. However, more information on nanoparticles containing neuroprotective agents with stem cell transplantation would help us to establish a greater degree of accuracy on this matter.

However, we have some limitations in this study. First, we compared water content only in the whole brain and we did not measure the permeability of the BBB after TBI. Second, we did not compare niosomal nanoparticles without curcumin with CM-NPs in the current study. However, the efficacy of niosomal nanoparticles without curcumin in comparison with CM-NPs was assessed on behavioral tests by our team in another study. Our data showed that niosomal nanoparticles without curcumin didn’t have any beneficial effects compared to the CM-NPs group. However, it should be noted that our results indicated the beneficial efficacy of CM-NPs in combination with NSCs in the context of TBI.

In conclusion, the most obvious finding to emerge from this study is that the general locomotor activity is improved in rats treated with the combination of hNS/PCs and CM-NPs. One of the more significant findings to arise from our study is that CM-NPs and hNSCs transplant appear to be an effective treatment to alleviate TBI-induced brain edema. The third major finding is that the number of reactive astrocytes significantly decreased when treated with hNSCs in conjunction with CM-NPs. This study has also found that monotherapy with hNSCs or CM-NPs is an effective treatment to modulate the TLR4/NF-κB signaling pathway in the course of TBI. However, future investigation on the fate of stem cells after transplantation in combination with CM-NPs would help us to have a comprehensive view of the efficacy of this method. Taken together, nano-drug delivery systems and neural stem cell therapy may have the potential to offer new therapeutic availability and efficacy over usual treatment.

## Materials and methods

### Study design

**Figure 8:**
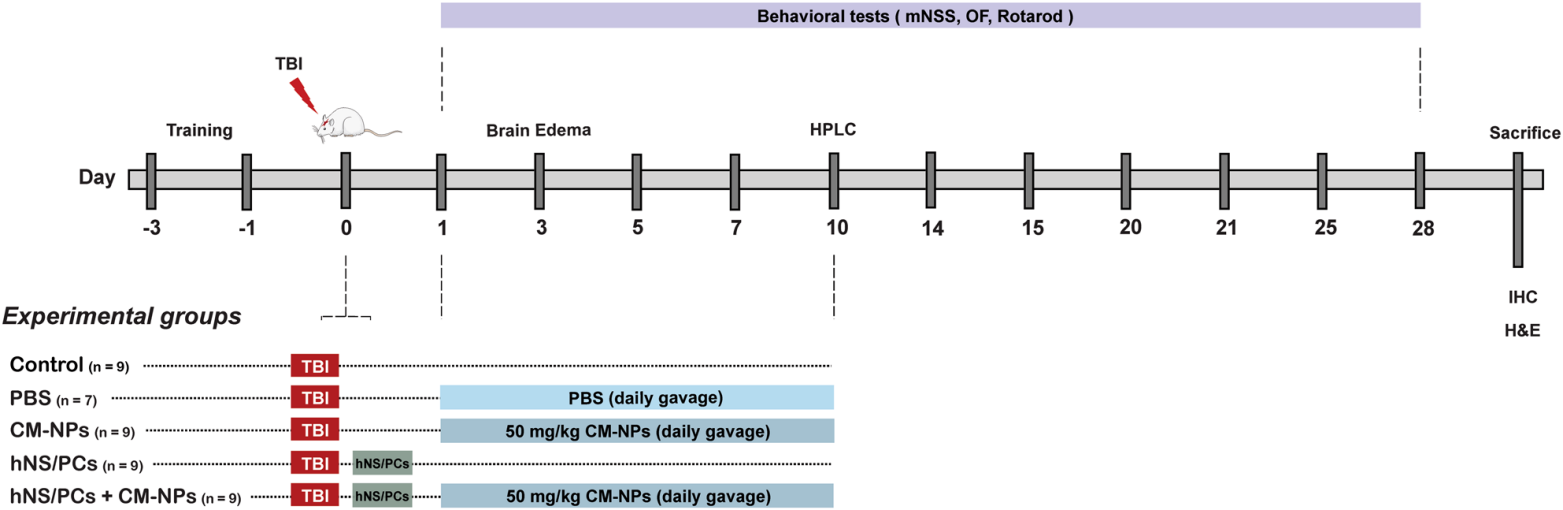
An overview of experimental design. Rats were trained 3 days before TBI to adapt to behavioral tests. Stem cell transplantation was performed 10 min after TBI in rats. The rotarod test was performed before TBI as well as on 5, 10, 15, 20, and 25 days, open field (OF) test was performed on 7, 14, 21, and 28 days, and mNSS test was performed Pre-TBI as well as 1, 7, 14, 21, and 28 days after TBI. In the CM-NPs and hNS/PCs + CM-NPs groups, 50 mg/kg CM-NPs were dissolved in PBS and gavaged daily. To evaluate brain water content, the brains were weighed on day 3 after TBI and incubated at 100 °C for 24 h and then were reweighed. In addition, HPLC analysis was performed to find out the optimal dose of CM-NPs on day 10 after TBI. After a 28-day treatment period, rats were sacrificed to assess lesion volume, gliosis, and TLR4/NF-κB inflammatory pathway by HE staining and IHC method, respectively.

NS/PCs were isolated from the human fetal brain and cultured in the neural basal medium. To evaluate the effects of combining hNS/PCs with CM-NPs, Wistar rats were subjected to moderate TBI using a biopsy punch. As shown in Fig. 8, behavioral assessments (i.e., modified neurological severity scores (mNSS), open field (OF), and rotarod) were performed at predetermined time points. To figure out the functional improvement, rats were sacrificed under anesthesia and brain tissue was assessed by histochemistry and immunohistochemistry (IHC).

### Animal care and ethical statement

Forty-five male Wistar rats (200 ± 20 g) were purchased from the Animal Center of Mashhad University of Medical Sciences. They received humane care in compliance with the guidelines of the ethics of laboratory animals care and the international norms and standards for conducting medical research. Animals were kept under a 12 h light/dark cycle in 30–70% humidity and constant temperature (22–24 °C) environment with ad libitum access to food and water. To eliminate the anxiety from human contact, the rats underwent adaptation exercises before performing behavioral tests. Also, some of the experiments were performed by an investigator who was blinded to the experimental groups to avoid any possible biases.

### Anesthesia and surgical procedures

Rats were anesthetized with an intraperitoneal (i.p.) injection of ketamine (80 mg/kg) and xylazine (20 mg/kg). After shaving and disinfection of the head area, the rat's skull was fixed in a stereotactic frame. TBI model was done as previously described^56^. Briefly, after incising the skin and retracting the fascia with a sterile blade, a square-shaped part of the skull was removed (AP = from − 1.5 to + 1.5 mm; ML = from − 0.2 to − 3 mm) using a dental micro drill. A punch biopsy (2 mm diameter) was attached to the drill and removed a small portion of the brain (AP = 0 mm; ML = − 1.5 mm; DV = − 2 mm: M1/M2 area). After cell implantation (cell recipient groups), the skin was closed with sutures.

### Experimental groups and treatments

Animals were randomly divided into five groups (Fig. 8) and TBI was done in all animals of experimental groups. No treatment was performed in the control group. PBS, hNS/PCs, CM-NPs, and hNS/PCs + CM-NPs groups were treated by PBS (vehicle), hNS/PCs, CM-NPs, and hNS/PCs + CM-NPs, respectively. The live cells (~ 5 × 10^5^) were diluted in PBS (10 μl) and then transplanted 10 min after TBI by a Hamilton syringe into the site of injury for hNS/PCs and hNS/PCs + CM-NPs groups. The CM-NPs were dissolved in PBS and administered by oral gavage (50 mg/kg daily) in CM-NPs and hNS/PCs + CM-NPs groups after TBI for 10 days. In the PBS group, the animals received PBS by oral gavage for 10 days after TBI. During the experiments, animals were checked for mortality/viability daily and only 2 of 45 rats died after the injury.

### Cell culture

In this study, NS/PCs were isolated from the human fetal brain in Brain Bio-bank of Neuroscience Department, Faculty of Medicine, Mashhad, Iran. The expansion of NS/PCs was performed as previously described^22^. Briefly, the hNS/PCs were taken from liquid nitrogen. The cells were gradually thawed by flipping up and down the medium containing fetal bovine serum (FBS) and then centrifuged (1500 rpm for 5 min). The supernatant was discarded and cells were transferred into 25 cm^2^ flasks containing Dulbecco's modified essential medium/F12 (DMEM/F12) (Gibco, Germany) with 1% glutamine (Gibco, Germany), 20 ng/ml epidermal growth factor (EGF) (Sigma, Germany), 1% Penicillin/Streptomycin solution (Gibco, Germany), 1% N2 supplement (Gibco, Germany), 1% B27 supplement (Gibco, Germany), and kept in a humid 37 °C incubator (5% CO_2_). This medium was recovered after 24 h of incubation and then changed every 3–4 days with a fresh medium. When the cells reached ~ 80% confluence, adherent cells were trypsinized and passaged. Expanded cells were used for transplantation when they reached the required number (About 5 × 10^5^ cells per animal).

### Characterization of NS/PCs

Characterization of human embryonic NS/PCs was performed previously^57^. To identify the stemness property, immunocytochemistry against nestin antibody as a neural stem cell marker (1:200, Sigma, Germany) was used. Fluorescein isothiocyanate (FITC) goat anti-rabbit (1:1000, Abcam, USA) was used as the secondary antibody. Then, the nuclei were counterstained by propidium iodide (PI)-(1:1000, Sigma, Germany). The primary antibody was omitted as a negative control. Finally, to visualize the marker, images were taken by invert microscopy (Axiovert-200, Ziss, Germany).

### Preparation of CM-NPs

Curcumin-loaded niosome nanoparticles (CM-NPs) were obtained from Food Science and Technology Research Institute, Mashhad, Iran. CM-NPs were prepared using the thin-film hydration method that curcumin was encapsulated in the shell of niosome nanoparticles and the characteristics of CM-NPs were described previously^29^.

### Ethics approval and consent to participate

All scientific procedures of this study have been approved by the Research Ethics Committee of Mashhad University of Medical Sciences (IR.MUMS.REC.1399.166) and performed in accordance with the ARRIVE guidelines. The NS/PCs used in this study were isolated from the human fetal brain. The experimental protocols were approved by the Brain Bio-Bank of Neuroscience Department (Faculty of Medicine, Mashhad, Iran) accordance with the ethical standards of the responsible committee on human experimentation (World Medical Association Declaration of Helsinki 2000). Written consent was obtained from the donors' parents and it was confirmed that no organs/tissues were procured from prisoners.

## Behavioral assessments

### Rotarod test

To evaluate high‑level loco-motor scores including long‑term weight support and hind-limb coordination, the rotarod test was used^58^. The painstaking description of the rotarod test was described previously^59^. Briefly, in this study, the time that animals can stay on an accelerating rotarod cylinder was measured. To get unbiased data, the mean latency to fall off three measurements (in seconds) on the rotarod was recorded at predetermined time points (i.e., at 5, 10, 15, 20, and 25 days compared with pre-TBI records). To remove odor, the rotarod cylinder was washed with 70% ethanol between each trial.

### mNSS test

To assess the sensory-motor functions, modified Neurological Severity Scores (mNSS) test was performed. The test is rated on an 18-point score, in which the normal score is 0 and the maximal deficit score is 18. A score of 13–18 indicates severe injury, 7–12 represents moderate injury, and 1–6 shows mild injury^60^. In our study, mNSS was performed at predetermined time points (i.e., before TBI and 1, 7, 14, 21, and 28 days after TBI) by an investigator who was blinded to the experimental groups.

### Open field test

The open field (OF) test was used to assess anxiety-like and exploratory behaviors at 7, 14, 21, and 28 days after TBI. All rats underwent adaptation to the testing environment. Predominantly, OF is rectangular that is surrounded by walls. The animal was placed in the center of OF and their total distance traveled (m) was recorded by a video camera for 10 min. Each trial was recorded and analyzed using Tracking software (Borj-Sannat, Iran)^56^. The arena was cleaned with 70% ethanol between each trial to remove odor.

### Study of achieving the optimal dose of CM-NPs

To find the optimum dose of CM-NPs, an in vivo study was designed similar to the main study. Thirty-six male Wistar rats (200 ± 20 g) were subjected to TBI and divided randomly into 4 groups. Oral gavage of different doses of CM-NPs (i.e., 25, 50, and 100 mg/kg) was dissolved in PBS and gavaged orally for 10 days after TBI. Three behavioral tests, such as rotarod, mNSS, and OF were performed at predetermined time points similar to the main study for 4 weeks. Following fulfilling behavioral tests, the most effective dose of CM-NPs was calculated by analyzing the results for the present study.

### HPLC test

To evaluate the ability of CM-NPs to cross the BBB, High-performance liquid chromatography (HPLC) was performed by measuring the concentration of curcumin within the brain. Rats in treatment groups received 25, 50, and 100 mg/kg of body weight CM-NPs. All concentrations of CM-NPs were administered orally at a daily dose for 10 days. A group received PBS as a control. Rats were sacrificed after administration of a mixture of ketamine (80 mg/kg, i.p) and xylazine (20 mg/kg, i.p) anesthesia to determine the distribution of the CM-NPs in the brain. Samples of the brains were rapidly removed and kept at − 80 °C before the examination. Three samples of the hippocampus, striatum, and lesion area were isolated from the injured hemisphere of the brain and placed on an ice bed. For the negative control, the brain tissue of the PBS group was used. All samples were homogenized in PBS, vortexed for 10 min, and then centrifuged at 10,000 rpm for 10 min at 4 °C. Finally, the sample was injected into the HPLC system^61^. To this point, in the HPLC system (Waters, USA) a binary pump with a UV detector and reverse phase (particle size 5 μm) was used for chromatographic separation. The mobile phase contained 5% acetonitrile solution in water buffered (pH 2.7) at 1 mL/min flow rate^62^.

### Measurements of brain edema

The percent of tissue swelling was calculated by Brain water content at 72 h after TBI. To this purpose, three rats from each group were sacrificed under anesthesia with ketamine (80 mg/kg, i.p) and xylazine (20 mg/kg, i.p), and their brains were removed immediately and divided into two hemispheres along the midline. Ipsilateral and contralateral hemispheres separately were placed on a piece of aluminum foil to obtain the wet weight and then dried in an electric oven at 100 °C for 24 h. Then, the samples were weighted to obtain dry weight. The percent of brain water content was calculated as follows^63^:

$$\% {\text{Water content}} = {1}00 \times \left( {{\text{Wet weight}} - {\text{Dry weight}}} \right)/\left( {\text{Wet weight}} \right).$$

### Tissue preparation

To prepare tissues for histochemistry and immunohistochemistry, rat brains were removed and fixed in 10% buffered neutral formalin (BNF). Tissue processing comprises several stages that cause structural arrangements of the tissues allowing them to be sectioned and hence stained. The dehydration process of samples was performed by passing them through ethanol containers with descending gradient. Also, to increase the tissue penetration of paraffin, the samples were passed through xylene containers. Finally, rat brains were embedded in paraffin blocks and stored at room temperature^64^.

### Assessment of the lesion volume

To evaluate the lesion volume, a series of coronal sections (5 μm thick) were cut and collected at 100 μm intervals (n = 10 sections for each rat). After that, the sections were air-dried at room temperature (22–25 °C) and stained with hematoxylin–eosin (HE). The images were captured by a light microscope (Olympus BX51, Japan) and analyzed by Image J software. The lesion volume was estimated by the following equation:

$${\text{Lesion volume}} = 0.{\text{5D}}\left( {{\text{A1}} + {\text{An}}} \right) + {\text{D}}\left( {{\text{A2}} + {\text{A3}} + \cdots + {\text{An}} - {1}} \right)$$

D is the distance between sections and A is the area of the cavity in the relevant section^15^.

### Immunohistochemistry

Immunohistochemistry on paraffin-embedded coronal sections (5 μm thick) was performed as described previously^40^. Briefly, after deparaffinization and dehydration, sections for antigen retrieval were kept in boiling PBS for 20 min (pH 7.4). Bovine serum albumin (BSA) for blocking background staining and 3% H_2_O_2_ in methanol for blocking endogenous peroxidase enzyme were used. Normal goat serum was used as a blocking solution (20 min at room temperature) and then exposed to different primary antibodies, including rabbit anti-glial fibrillary acidic protein (GFAP) a marker of astrocytes (1:2000; ab7260, Abcam, USA); rabbit anti-ionized calcium-binding adapter molecule 1 (Iba1) a marker of microglia (1:1000; LKG5732, Wako, USA); rabbit anti-TLR4 antibody (1:500; ab217274, Abcam, USA), rabbit anti-NF-κB p65 antibody (1:1000; ab16502, Abcam, USA), and rabbit anti- TNF-α antibody (1:100; NBP1-19532, Novus, USA) at 4 °C overnight. After washing the sections, the secondary antibody goat anti-rabbit HRP (1:500; ab6721, Abcam, USA) was applied at room temperature for 90 min. Then, diaminobenzidine (DAB) chromogen was used for light microscopy. Also, hematoxylin was used to counterstain cell nuclei. After dehydration and clearing, the sections were sealed with neutral gum (Entellan, Merck, Germany). For negative controls, primary antibodies were discarded. The sections were evaluated with a bright field microscope (Olympus 30X23, Japan). GFAP, Iba1, TLR4, NF-kB, and TNF-α positive cells were used for statistical analysis. Positive cells were counted in 5 fields of microscope ocular lens (40×) at the edge of the cavity. The mean number of positive cells in each group was presented as the number/lesion area.

### Statistical analysis

Analyses were conducted by GraphPad Prism software (version 6.01). Statistically significant differences between various groups were assessed using a one-way analysis of variance (ANOVA). Post hoc multiple comparisons were conducted by Tukey's tests. Statistical analysis of the rotarod, mNSS, and OF tests between groups on different days was performed by two-way repeated-measure ANOVAs with Tukey's post hoc tests. Data were presented as the mean ± standard error of the mean (SEM) and the significance level was considered at *P* < 0.05.

## Data Availability

The data that support the findings of this study are available from the corresponding author upon reasonable request.
